# Seroprevalence and molecular characteristics of varicella-zoster virus infection in Chinese children

**DOI:** 10.1186/s12879-019-4233-7

**Published:** 2019-07-19

**Authors:** Lin Luan, Xiaochen Shen, Jing Qiu, Yang Jing, Jingqi Zhang, Jie Wang, Jun Zhang, Chen Dong

**Affiliations:** 1 Suzhou Center for Disease Control and Prevention, 72 Sanxiang Road, Suzhou, 215000 China; 2 Gusu Center for Disease Control and Prevention, Suzhou, China; 3 Department of Epidemiology and Statistics, School of Public Health, Jiangsu Key Laboratory and Translational Medicine for Geriatric Disease, Medical College of Soochow University, 199 Renai Road, Suzhou, 215123 Jiangsu China; 4 Shanghai Center for Disease Control and Prevention, Shanghai, China

**Keywords:** Varicella-zoster virus, Seroprevalence, vaccination, Genotype

## Abstract

**Background:**

Varicella-zoster virus (VZV) infection in children is an important public health problem in China. We performed the current study to explore the seroprevalence of VZV infection in Chinese children in order to provide more information for improvement of varicella vaccination in China.

**Methods:**

Three thousand fourteen children were recruited from Chinese kindergarten students aged from four to six years. Anti-VZV IgG and IgM were assayed using enzyme-linked immunosorbent assay. Both ORF22 and ORF62 of VZV were amplified, sequenced, and analyzed by nested PCR.

**Results:**

Among 3014 children, 43.9% of boys and 46.3% of girls were vaccinated with varicella vaccine, respectively. The seroprevalence of anti-VZV IgG was 54.4% in the children with varicella vaccination, which was significantly higher than those in unvaccinated children (49.2%) (χ^2^ = 8.206, *P* = 0.004). Among of the vaccinated children, the detection rates of VZV IgG antibody increased with age, with 49.4, 50.9 and 58.9% in 4, 5 and 6-year groups, respectively (Trend χ^2^ = 17.202, *P* = 0.002). However, there was no difference in anti-VZV IgG detection rates among those unvaccinated children in different age groups (Trend χ^2^ = 8.681, *P* = 0.070). In addition, 13 boys and 13 girls were positive for anti-VZV IgM, respectively. Among of them, eight children (0.6%) have received varicella vaccination, which was similar to those in unvaccinated children (1.1%). However, only one ORF22 sequence was isolated from an unvaccinated 5-year boy. Compared to the reference VZV sequences, the nucleotide homology was estimated to be 99.7% with genotype J.

**Conclusions:**

Our study indicated that about half of Chinese children aged four to six years have a high risk of VZV infection. It should be helpful for the evaluation on the necessity of varicella immunization in China.

## Background

Varicella-zoster virus (VZV), a member of the family *Herpesviridae* and the subfamily *Alphaherpesvirinae*, is usually transmitted by airborne droplets or close personal contact [1–3]. The primary VZV infection mainly occurs in childhood and causes varicella (chickenpox), which has generally mild and easily diagnosed syndromes. However, an estimated 2–6% of varicella cases can develop complications including bacterial superinfections as well as neurologic or pulmonary disorders [4, 5]. Following primary infection, the virus may become latent in the dorsal root ganglia, cranial nerve ganglia or autonomic ganglia and can reactivate, causing herpes zoster (HZ) or ‘shingles’, which typically occurs in people aged more than 50 years [6–8].

Safe and effective live, attenuated varicella vaccine was developed in 1974, which was produced by a seed virus isolated from a Japanese boy (named OKA) with typical varicella. Before the introduction of varicella vaccine, the disease was very common worldwide [9, 10]. With increasing vaccine coverage in the countries that implemented universal varicella vaccination for children, the incidence of varicella significantly decreased by 80–85% after a single dose and by 98–99% after a second vaccine dose compared with the pre-vaccination era, respectively [11]. Moreover, varicella-related hospitalization and mortality rates dramatically decreased by 75–88% and 88–97% after a single dose of vaccine, respectively [12]. Despite that varicella vaccine is recommended by the World Health Organization (WHO) and is available throughout the world, it has only been incorporated into the national immunization program for children in a small number of countries because it is relatively expensive for the developing regions [13].

VZV infection is an important public health problem in China. More than 90% of varicella cases have been reported in the children aged less than 15 years [14, 15], and about 5% of these cases develop severe complications [16], especially in kindergarten children. Although several Chinese epidemiological surveys have suggested that varicella vaccination could decrease the disease burden, the vaccine is not mandatory for children to date [11, 17–19]. In order to provide additional information for improvement of varicella vaccination strategies in China, we performed the current population-based study to analyze the seroprevalence of VZV infection in Chinese kindergarten children with or without varicella vaccination.

## Methods

### Study population and sample collection

Between August 2017 and September 2017, 3014 kindergarten students aged 4–6 years in Suzhou Industrial Park (Jiangsu, China) were invited to participate in the present study when they received an annual physical examination. Written informed consents were obtained from the parents of children after declaring the aims of the present study. A blood sample (5 mL) was taken from 3003 children because 11 children did not agree to provide their blood samples. The samples were immediately spun down and the serum was stored at − 40 °C for later analysis. Varicella vaccination information including the date of administration and trademark of vaccine was retrieved from the Immunization Registry System or the detailed vaccination records. The study protocol was approved by the ethics committees of Suzhou Centers for Disease Control and Prevention in accordance with the ethical guidelines of the 1975 Declaration of Helsinki.

### Serological assessments

The serum samples obtained from all children were tested for anti-VZV IgG and IgM antibody by enzyme-linked immunosorbent assay (ELISA) with the commercial kits of “Diagnostic Kit for IgG Antibody to Varicella-Zooster Virus” and“Diagnostic Kit for IgM Antibody to Varicella-Zooster Virus” (Beier Biotechnology, Beijing, China), respectively. The cut-off value was calculated for each plate according to the manufacturer’s instructions. When an ambiguous result was found, the sample was retested and confirmed as positive only if one of the repeats (2/3 of the total tests) was positive. According to the manufacture’s introductions, both sensitivity and specificity of IgG method and IgM method were more than 98%, and the CV% of two methods were less than 15%.

### VZV DNA isolation and sequence analysis

VZV DNA was extracted by Viral RNA/DNA Extraction Kit Version 5.0 (TaKaRa Biotechnology, Dalian, China) from 300 μL of serum. According to the previous reports [20, 21], a 359-bp region of the ORF22 gene (nt37870–nt38228, reference strain Dumas, XO4370) and a 419-bp region of ORF62 gene (nt106983–nt107401, reference strain Dumas, XO4370) were amplified by polymerase chain reaction (PCR). The primers used for ORF22 were ORF 22F: 5′-TAGCATGTCTGGAGGCAATGG and 22R: 5′-GGCCTTGGAAACCACATGATCG; and the primers used for ORF62 were ORF 62F: 5′- GGCCTTGGAAACCACATGATCG and 62R: 5′- CGTCTCCCGTTCCGCATGTAG. The PCR products were purified and sequenced using the ABI 3730 (Applied Bio systems, Foster City, USA). The genotype of isolated VZV strain was identified using phylogenetic analysis by the neighbor-joining algorithm in MEGA version 4.1 software. The reliability of the phylogenetic analysis was tested by bootstrap analysis with 1,000 replicates. VZV reference wild-type sequences of genotype E strains included Dumas, BC (AY548171) and SD (DQ479953), genotypes E2 strains included HJO (AJ871403) and 11 (DQ479955), genotype M1 strain CA123 (DQ457052), genotype M2 strains included DR (DQ452050) and 8 (DQ479960), and genotype J strains included pOka (AB097933), pOka-derived vaccine strain (vOka; AB097932), VariVax (DQ008355) and Varilrix (DQ008354) were used to identify nucleotide variability.

### Statistical analysis

Statistical analysis was performed using SAS version 9.2 software (SAS Institute Inc., Cary, USA). The seroprevalence of anti-VZV IgG antibody between different groups were compared using the χ^2^ test or trends χ^2^ test. All tests were two sided, and differences were considered statistically significant if *P* < 0 05.

## Results

### Vaccination characteristics of studied Chinese children

**Fig. 1:**
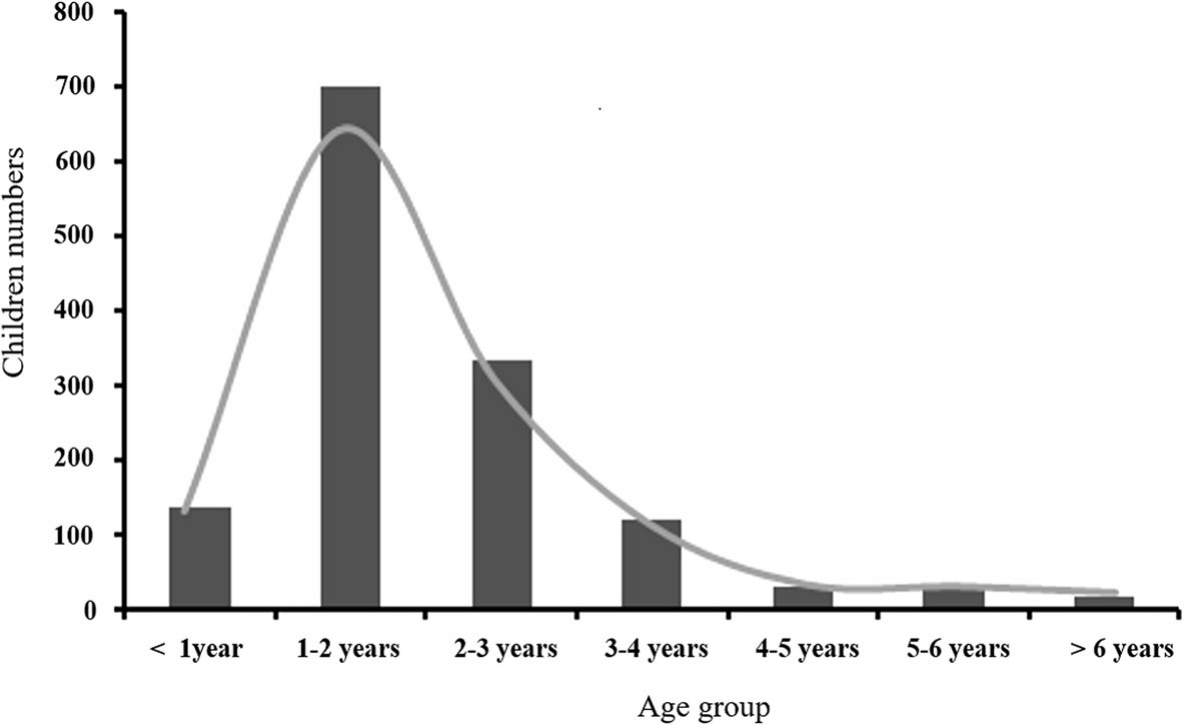
The age distribution of the Chinese children administered with the first dose of varicella vaccine. The bars represented the number of the children with the first dose of varicella vaccine in different age groups. The lines showed the trend of the age distribution of the children with the first dose of varicella vaccination

Among 3014 children, 701 boys (43.9%) and 656 girls (46.3%) were vaccinated with Vari-L varicella vaccine (Changchun Institute of Biology Products, Changchun, China), respectively, and only 21 children received the second vaccine dose. The mean ages of the first and the second dose of vaccine were 5.20 ± 0.83 and 5.71 ± 0.46 years, respectively. A total of 33.7, 45.6 and 53.6% of children were vaccinated in the 4, 5 and 6-year groups, respectively (Trend χ^2^ = 81.678, *P* = 0.000, Table 1). Among of them, 137 (10.1%), 699 (51.5%), 331 (24.4%), 118 (8.7%), 29 (2.1%), 26 (1.9%) and 17 (1.3%) children were administered with the first dose of vaccine at < 1, 1–2, 2–3, 3–4, 4–5, 5–6 and ≥ 6 years of age, respectively (Fig.1).

**Table 1:** Characteristics of varicella vaccination among Chinese Children aged 4 to 6 years

	Varicella vaccinated (%)	Varicella unvaccinated (%)	χ^2^	*P*
Total				
Boys	701 (43.9)	896 (56.1)	1.747	0.187
Girls	656 (49.8)	761 (50.2)		
4 years				
Boys	163 (32.3)	342 (67.7)	0.922	0.337
Girls	147 (35.5)	267 (64.5)		
5 years				
Boys	211 (43.4)	275 (56.6)	1.691	0.193
Girls	221 (47.8)	241 (52.2)		
6 years				
Boys	327 (54.0)	279 (46.0)	0.061	0.813
Girls	288 (53.2)	253 (46.8)		
Trend χ^2^ = 81.678, *P* = 0.000

### Seroprevalence of anti-VZV IgG among studied children

In the present study, ELISA was performed on the serum samples of the 3003 participants (1592 boys and 1411 girls). As the results shown, 837 boys (52.6%) and 710 girls (50.3%) were positive for anti-VZV IgG, respectively (χ^2^ = 1.776, *P* = 0.411). The seropositivity of VZV IgG increased with age, which was detected in 47.6, 49.2 and 55.5% of the children in 4, 5 and 6-year groups, respectively (Trend χ^2^ = 27.071, *P* = 0.000).

Among of 1357 children with varicella vaccination, the seroprevalence of anti-VZV IgG was 54.4%, which was significantly higher than those in unvaccinated children (49.2%) (χ^2^ = 8.206, *P* = 0.004. Table 2). Additionally, among of the vaccinated children, the detection rates of VZV IgG antibody increased with age, with 49.4, 50.9 and 58.9% in 4, 5 and 6-year groups, respectively (Trend χ^2^ = 17.202, *P* = 0.002). However, there was no difference in anti-VZV IgG detection rates among unvaccinated children, with 46.7, 49.4 and 51.7% in 4 years, 5 years and 6-year groups, respectively (Trend χ^2^ = 8.681, *P* = 0.070) (Table 2).

**Table 2:** Seroprevalence of anti-VZV IgG antibody among vaccinated and unvaccinated children aged 4 to 6 years

	Vaccinated		Unvaccinated		χ^2^	*P*
	IgG Positive (%)	IgG Negative (%)	IgG Positive (%)	IgG Negative (%)		
Total	735 (54.4)	616 (45.6)	812 (49.2)	840 (50.8)	8.206	0.004
4 years	153 (49.4)	156 (50.6)	284 (46.7)	324 (53.3)	0.538	0.463
5 years	220 (50.9)	207 (49.1)	253 (49.4)	259 (50.6%)	0.334	0.563
6 years	362 (58.9)	253 (41.1)	275 (51.7)	257 (48.3)	5.652	0.017
Trend χ^2^ = 17.202, *P* = 0.002			Trend χ^2^ = 8.681, *P* = 0.070			

The results from subgroup analysis showed that among vaccinated children, the seroprevalence of anti-VZV IgG in 5 years old boys was significantly higher than those in 5 years old girls (55.0% vs 48.1%, χ^2^ = 6.823, *P* = 0.033). However, no difference in VZV IgG antibody seropositivity was detected between unvaccinated boys and girls with 5 years of age. Additionally, no significant difference in anti-VZV IgG detection rates was detected between boys and girls in 4- and 6-year groups regardless of vaccination status (Table 3).

**Table 3:** Subgroup analysis of anti-VZV IgG antibody among vaccinated and unvaccinated children aged 4 to 6 years

	Vaccinated		χ^2^	*P*	Unvaccinated		χ^2^	*P*
	IgG Positive (%)	IgG Negative (%)			IgG Positive (%)	IgG Negative (%)		
Total								
Boys	387 (55.3)	313 (44.7)	3.410	0.182	450 (50.4)	442 (49.6)	1.303	0.257
Girls	348 (53.5)	303 (46.5)			362 (47.6)	398 (52.4)		
4 years								
Boys	78 (48.1)	84 (51.9%)	1.159	0.560	166 (48.5)	176 (51.5)	1.137	0.289
Girls	75 (51.0)	72 (49.0)			118 (44.2)	149 (55.8)		
5 years								
Boys	116 (55.0)	95 (45.0)	6.823	0.033	133 (48.9)	139 (51.1)	0.825	0.662
Girls	104 (48.1)	112 (51.9)			120 (50.0)	120 (50.0)		
6 years								
Boys	193 (59.0)	134 (41.0)	0.007	0.935	151 (54.1)	128 (45.9)	1.387	0.259
Girls	169 (58.7)	119 (41.3)			124 (49.0)	129 (51.0)		

### Seroprevalence of anti-VZV IgM among studied children

Twenty-six children (13 boys and 13 girls) were positive for anti-VZV IgM. Among of them, eight childrenhave received varicella vaccination, which was similar to those in unvaccinated children (0.6% vs 1.1%, χ^2^ = 2.153, *P* = 0.142). Additionally, the seropositivity of anti-VZV IgM was 0.5, 1.1 and 1.0% in 4, 5 and 6-year groups. There was no significant difference in the detection rates of VZV IgM antibody among three groups (Trend χ^2^ = 1.625, *P* = 0.444).

### Analysis of VZV DNA amplified from children

**Fig. 2:**
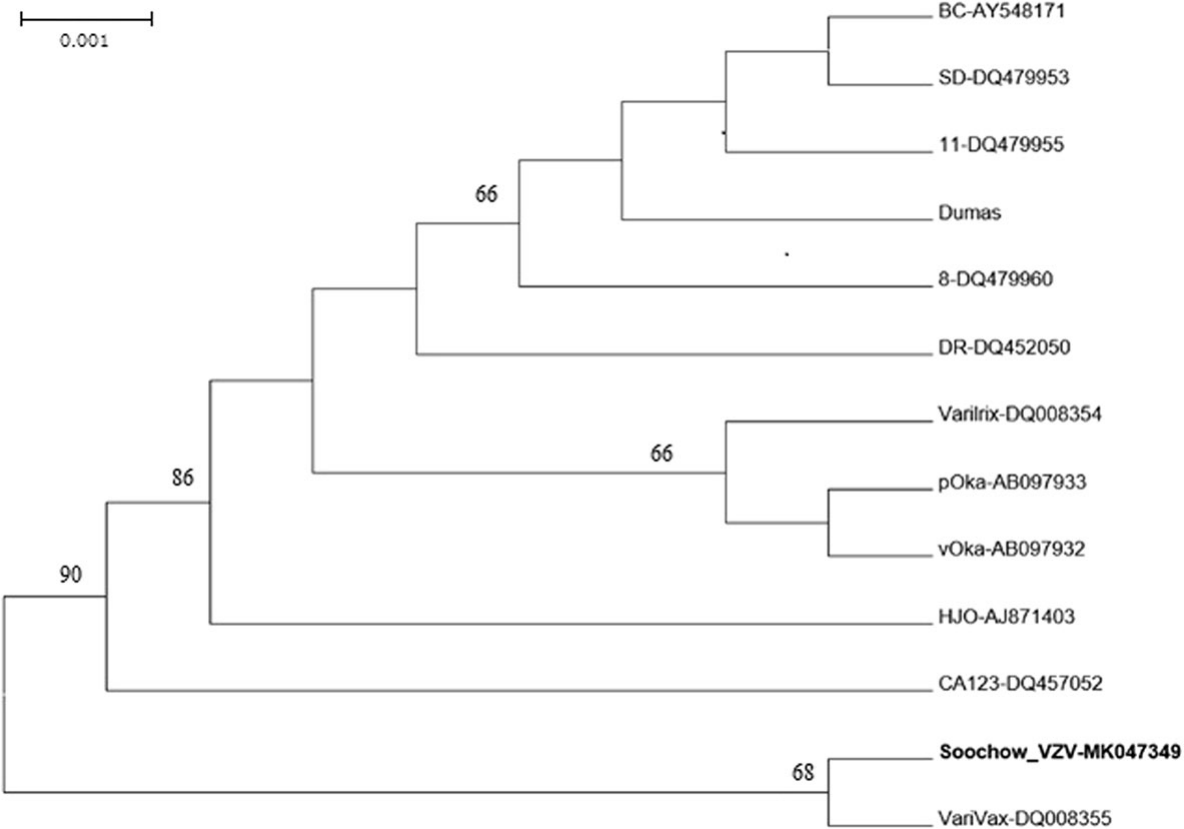
Phylogenetic analysis based on nucleotide sequencing of the 359 bp ((nt37870–nt38228) of ORF22 region. The strain Soochow_VZV of genotype J was isolated from the present study. VZV reference wild-type sequences of genotype E strains included Dumas, BC (AY548171) and SD (DQ479953), genotypes E2 strains included HJO (AJ871403) and 11 (DQ479955), genotype M1 strain CA123 (DQ457052), genotype M2 strains included DR (DQ452050) and 8 (DQ479960), and genotype J strains included pOka (AB097933), pOka-derived vaccine strain (vOka; AB097932), VariVax (DQ008355) and Varilrix (DQ008354) were used to identify nucleotide variability. Percent bootstrap support (values > 65%) was indicated at the respective nodes

Twenty-six anti-VZV-IgM-positive samples were further analyzed to determine the existence of VZV DNA by amplification of 359-bp ORF22 sequence and 419-bp ORF62 sequence, respectively. However, only one ORF22 sequence was obtained from an unvaccinated 5-year boy. Compared to the reference VZV sequences, the nucleotide homology was estimated to be 99.7% with genotype J, but only 98.6, 98.6, 98.9 and 99.4% similar to the genotype E, E2, M1 and M2 VZV strains, respectively. Therefore, this VZV isolate, named Soochow_VZV (GenBank no. MK047349) was classified as genotype J (Fig. 2), which was widely distributed in eastern China.

## Discussion

This study was conducted to analyze the seroprevalence of VZV infection in children with the aim to provide additional data for the evaluation of universal varicella vaccination program in China. As expected, the results showed that the seropositivity of anti-VZV IgG in vaccinated children was significantly higher than those in unvaccinated children. Moreover, the seroprevalence of anti-VZV IgG increased with age among vaccinated children (49.4% in age 4, 50.9% in age 5 and 58.9% in age 6). Previous results from a Korean study reported that the anti-VZV IgG seropositivity was 49% in age 4, 62% in age 5 and 70% in age 6, respectively [22]. The detection rate of VZV IgG antibody in Chinese vaccinated children was obviously lower than those in Korean vaccinated children. Considering that Korea has already incorporated varicella vaccine in the National Immunization Program, more scientific vaccination program is needed to be established and recommended to the children in China.

Among unvaccinated children, more than half of them were anti-VZV IgG negative. No significant difference in anti-VZV IgG seropositivity was detected among the 4, 5 and 6-year groups and between boys and girls. These results indicated that more than half of the children had a high risk of VZV infection. Therefore, the varicella vaccination coverage should be broadened in China. Although the varicella vaccine is a live, attenuated vaccine, several studies have indicated that a second dose of the vaccine would provide higher antibody levels [23–25]. Moreover, the Advisory Committee on Immunization Practice and German Standing Committee on Vaccination have recommended a second dose varicella vaccine because of the waning of immunity. Therefore, varicella vaccine can be recommended to the children even they have been infected by VZV or administered with the first dose of vaccine before.

VZV genotypes demonstrate a specific geographical distribution. Genotypes E1 and E2 are dominant in Europe [20]. Genotype M (including M1, M2, M3 and M4) is mainly detected in tropical regions in Africa and Central America [26, 27]. Genotype J is the most prevalent VZV in the east Asia [28]. In the present study, one genotype J VZV strain was detected from 26 samples with anti-VZV IgM positive. Similar to the present findings, Sun et al. identified the 69 VZV strains from clinical patients between May 2011 and June 2013 in eastern China as genotype J [29]. Therefore, our results and previous findings suggested that the current varicella vaccine is suitable for regular use in China because the vaccine strain (Oka strain) belongs to genotype J.

However, our study has certain limitations. First, the samples were collected from a single eastern Chinese city of Suzhou, and thus might not represent the whole China. Second, because of the low virus titers, the VZV strain isolated from this study was genotyped only based on ORF22 fragment but not whole genome or restriction fragment length polymorphisms. Last, our cross-sectional study just analyzed the seroprevalence data about VZV infection. Long-term follow-up studies should be performed on the high-risk children who are need vaccination.

## Conclusions

Our present study revealed that about half of children aged four to six years have a high risk of VZV infection in China. As such, varicella immunization is needed to prevent virus infection and transmission.

## Data Availability

The datasets generated during and/or analyzed during the current study are available from the corresponding author on reasonable request.

## Abbreviations

ELISA: Enzyme-linked immunosorbent assay
PCR: Polymerase chain reaction
VZV: Varicella-zoster virus
